# Yellow and the Novel Aposematic Signal, Red, Protect *Delias* Butterflies from Predators

**DOI:** 10.1371/journal.pone.0168243

**Published:** 2017-01-06

**Authors:** Jocelyn Liang Qi Wee, Antónia Monteiro

**Affiliations:** 1 Department of Biological Sciences, National University of Singapore, Singapore, Singapore; 2 Yale-NUS College, Singapore, Singapore; University of Sussex, UNITED KINGDOM

## Abstract

Butterflies of the South Asian and Australian genus *Delias* possess striking colours on the ventral wings that are presumed to serve as warning signals to predators. However, this has not been shown empirically. Here we experimentally tested whether the colours of one member of this diverse genus, *Delias hyparete*, function as aposematic signals. We constructed artificial paper models with either a faithful colour representation of *D*. *hyparete*, or with all of its colours converted to grey scale. We also produced models where single colours were left intact, while others were converted to grey-scale or removed entirely. We placed all model types simultaneously in the field, attached to a live mealworm, and measured relative attack rates at three separate field sites. Faithful models of *D*. *hyparete*, suffered the least amount of attacks, followed by grey-scale models with unaltered red patches, and by grey-scale models with unaltered yellow patches. We conclude that red and yellow colours function as warning signals. By mapping dorsal and ventral colouration onto a phylogeny of *Delias*, we observed that yellow and red colours appear almost exclusively on the ventral wing surfaces, and that basal lineages have mostly yellow, white, and black wings, whereas derived lineages contain red colour in addition to the other colours. Red appears to be, thus, a novel adaptive trait in this lineage of butterflies.

## Introduction

Noxious animals often advertise their unpalatability to predators via the use of warning signals, also called aposematic signals, in the form of bright conspicuous colouration [1]. Aposematic signals have evolved independently in a wide range of vertebrate and invertebrate taxa [2–9], but their origin is explained by similar mechanisms. Naïve predators of aposematic species learn to form an association between warning colours and unpalatability through repeated exposure to the aposematic prey [10]. Aposematic colours lead to rapid recognition of distasteful prey, minimizing predators’ wasted predation attempts, and leading to increased prey survival [11].

For optimal learning of an aposematic signal, the signal should be (i) easily detected by potential predators, (ii) improve memory retention in the predator, and (iii) aid accurate recognition of prey so as to facilitate avoidance learning by predators [10]. Colours of warning signals per se, are believed to be essential in ensuring the efficacy of such signals, especially for avian predators, who have colour vision [12]. Aside from colours, other properties of visual warning signals have also been shown to enhance the efficacy of the aposematic display. For instance, high achromatic and chromatic contrast of warning signals [13] (by themselves or in contrast to the natural background), luminance contrast, and signal symmetry, have all been shown to affect the effectiveness of warning signals [14–16].

In addition to learning, the efficacy of a warning signal also depends on innate biases towards these signals. Past studies have shown that some predators, such as birds, display inherent innate aversion towards specific colours of aposematic prey. For instance, in a suite of experiments using naïve domestic chicks (*Gallus gallus domesticus*), the birds showed an innate aversion to red mealworms, preferring instead to feed on brown-coloured prey [17–19].

Despite the relative abundance of literature regarding aposematic signals, much uncertainty still remains as to which aspects of these signals are most critical in facilitating avoidance learning and memory retention in predators [15]. In addition, there is few information in the literature regarding the evolution of such signals in a close group of species. The most well studied group are the phenotypically polymorphic poison-dart frogs (family Dendrobatidae) whereby multiple studies have aimed to resolve the evolution of aposematic colours in relation to characters such as body size and toxicity levels using phylogenetic mapping methods [20, 21]. These studies revealed that dendrobatids have frequent shifts in colouration throughout their evolutionary history. This raises questions about the selective forces that are driving the evolution of body colouration as it has always been assumed that for aposematism to work efficiently, body colouration and visual markings should be relatively constrained to facilitate avoidance learning by predators [10].

Similarly to the frogs, *Delias* butterflies also display a remarkable array of colouration and patterns, with red and yellow being predominant colours within the genus. By investigating how colour variants affect the fitness of individuals, we can test whether colours have been evolving to serve an adaptive function. To date, however, there have been few comprehensive studies undertaken on detailed evolution of aposematic colours in butterflies. The most intensely studied mimetic butterflies belong to the genus *Heliconius*, with most studies focussing on the evolution of different colour pattern combinations within and between mimicry rings [22, 23]. For instance, both Kapan [24] and Langham [25] found that novel butterfly morphs displaying similar colours to the locally abundant morphs were predated at a higher frequency. This suggests that particular aposematic patterns within *Heliconius* species are being maintained by predator selection against novel morphs. Other studies, however, have shown that the coloured patterns of *Heliconious* butterflies alone are sufficient to confer protection against predators [24–27]. For instance, Finkbeiner et al. [28] found that coloured models of *Heliconius* butterflies were more effective in predator deterrence than achromatic models, which suggests that the colour aspect of warning signals is critical for predator deterrence. While there is some understanding of how aposematic colours developed within the *Heliconius* system, the origin of specific colours on the wings for these or other genera of butterflies, such as *Delias* butterflies, and their significance regarding how they might improve a prey’s general level of aposematism remain relatively unexplored.

**Fig 1:**
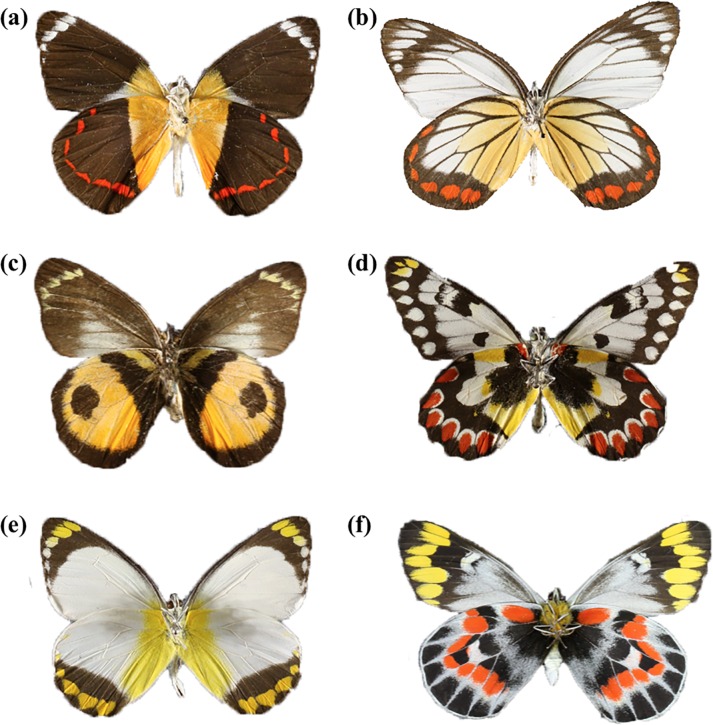
Examples of the ventral wing colouration found within *Delias*. (a) *Delias timorensis*, (b) *Delias hyparete luzonensis*, (c) *Delias albertisi*, (d) *Delias aganippe*, (e) *Delias ennia nigidius*, and (f) *Delias harpalyce*. (Source: Museum of Comparative Zoology, Harvard University).

Butterflies of the genus *Delias* (Hubner) belong to a widely distributed group of approximately 250 species throughout the Oriental, Southeast Asian, and Australian regions [29, 30]. As larvae, almost all species feed in a gregarious manner on mistletoes and host plants from Loranthaceae, Viscaceae and Santalaceae, and are thought to accumulate toxic chemical compounds from their host plants, making them distasteful to predators [31, 32]. Differing from other pierids, *Delias* adults possess bright colours on the ventral surface of their wings (shades of reds, oranges, and yellows contrasted with striking patterns of blacks), in particular on the hindwing (Fig 1). These bright colours have led some to believe that they function as an aposematic signal. In fact, many have proposed mimetic assemblages whereby *Delias* adults act as toxic models for Batesian mimicry due to remarkable similarities between *Delias* adults and other pierid species [33]. However, to this date, no experimental evidence is available to support both the proposed toxicity of the larval host plants [34], and the hypothesis that the colours of *Delias* species do indeed function as aposematic signals.

Not all conspicuous colouration serves as a defensive signal. Bright colours may also serve other functions, such as sexual signalling and thermoregulation [35–37]. For instance, the colour patterns of *Heliconius* butterflies are important in both predator deterrence and in mate choice [28, 35, 38]. Melanisation patterns on the wood tiger moths *Parasemia plantaginis*, have also been shown to have a significant contribution towards thermoregulation, with individuals with darker colouring warming up at a faster rate compared to less melanic individuals [39].

**Fig 2:**
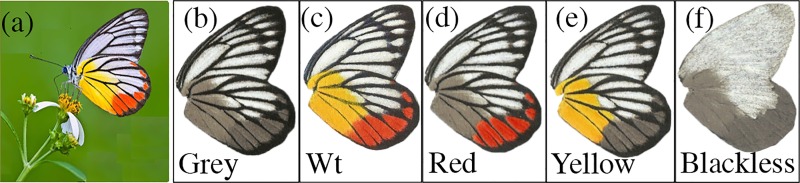
Artificial *Delias hyparete* models used across all trials. (a) the Painted Jezebel, *Delias hyparete* at rest, displaying its ventral wings (Credit: Sunny Chir). (a) Achromatic Grey model that served as the control model in every comparison. (b) Test pattern (Wt) resembling the wild-type colouration of *D*. *hyparete*. (c) Red model with the yellow component of the colouration converted to greyscale. (d) Yellow model with the red component of the colouration converted to greyscale. (e) Blackless model that resembles the achromatic control model, but with black venation pattern removed.

Therefore, to test the functional significance of the wing colours of *Delias* butterflies, we conducted a field-based study using artificial paper models with various colour manipulations of *Delias hyparete*, commonly known as the Painted Jezebel (Fig 2). This species is the sole representative of the genus *Delias* in Singapore. It is a common butterfly that is found throughout both forested and urban landscapes, and is characterised by its conspicuous yellow and red colours on its ventral wings contrasted with a black outline overlaying the veins [40]. Artificial butterfly models have been used successfully as experimental systems to study the function of both non-warning and warning visual colour signals [28, 38, 41–43]. Here we ask whether the bright colouration of *D*. *hyparete* functions as an aposematic signal, and we explore how the different colours of *D*. *hyparete* (red, yellow, black) affect the efficacy of the signal. We then reconstruct the origin of different colours, on both the dorsal and ventral surfaces on a phylogenetic tree of *Delias* species [30], to determine how aposematic colouration has evolved in this genus of butterflies.

## Materials and Methods

### (a) Experimental specimens

Eggs of *D*. *hyparete* were collected from their larval host plant, the Malayan Mistletoe (*Dendrophtoe pentandra)* found within the campus grounds of the National University of Singapore. The larvae were raised on leaf cuttings of the mistletoe in a climate-controlled room at 22°C with a 12:12hrs light:dark cycle and 65% relative humidity. Adult butterflies were frozen at -80°C on the day of eclosion to prevent any loss of scales due to wear and tear. Subsequently, hindwings were dissected for spectral measurements.

### (b) Preparation of artificial butterfly models

At rest, *D*. *hyparete* folds its wings over its body. The models were designed to display this natural resting position. We mostly used the methods outlined in Ho et al. [43] and Finkbeiner et al. [28] to produce the butterfly models. A digital photograph of *D*. *hyparete* (Fig 2A) was used and corrected for wingspan size using Photoshop CC 2014. The same software was used to modify wing colours on the ventral surface for the different paper models. Each image was duplicated and mirrored to produce identical left and right wings. A connecting rectangular band was added between the two images to hold a live mealworm to represent the body. Butterfly models were printed using an HP Deskjet 2540 printer with HP61 ink, on Whatman filter paper (Qualitative #1), which yields reflectance spectra that were similarly bright when compared to the real wings [28, 41]. Paraffin wax was then applied to all the models to render them weather-resistant. To ensure that accurate colours were used for the models, reflectance spectra measurements were taken from the three major wing colours: red, yellow, and white on the ventral side of *D*. *hyparete* using an Ocean Optics USD2000 fiber optic spectrometer. Each measurement was taken with the axis of the illuminating and detecting fibre directed at a 90° angle to the plane of the wing at a distance of 2mm using a deuterium-halogen tungsten lamp (DH-2000, Ocean Optics) as a standardised light source and calibrated using a white Ocean Optics WS-1 reflectance standard. If required, colours of the artificial models were corrected by filling in coloured regions of the model with colour pencils that had pigments that reflected similar wavelengths in terms of brightness and hue to the natural *D*. *hyparete* wings. The colour pencils used were: *Derwent C720* Coloursoft Pencil in White, *Derwent* 0600 Artists Pencil in Deep Cadmium (yellow), and *Prismacolor* Verithin Coloured Pencil 744 in Poppy Red. Final spectral measurements were taken for the finished models after the application of paraffin wax coating.

We produced five types of paper model: mimics of wild-type *D*. *hyparete* (Wt), as well as four other manipulated models: an achromatic model (Grey), (with all the colours on the wing converted to greyscale), a Red phenotype, a Yellow phenotype, and a Blackless phenotype, an achromatic model with the black vein colouration removed (Fig 2). The Grey model was used as a control against which all other colour variants were compared. Using the Wt model, we tested if experienced predators in the butterfly’s natural habitats would be deterred by the wild-type colouration of the Painted Jezebel. For the Red model, with the yellow component of the signal converted to greyscale, we tested the efficacy of red alone as a warning signal. For the Yellow model, with the red component of the warning signal converted to greyscale, we tested if yellow is sufficient on its own to deter predators. Lastly, for the Blackless model, with the black venation patterns removed from the achromatic model, we tested the function of black as a possible warning signal.

### (c) Avian colour vision modelling

Unlike humans, birds have tetrachromatic colour vision that is regulated by four classes of single-cone photoreceptors namely, long-wavelength sensitive (LWS), medium-wavelength sensitive (MWS), short-wavelength sensitive (SWS), and ultraviolet/violet sensitive (UVS/VS). Although peak sensitivities of LWS, MWS, and SWS photoreceptors are highly conserved amongst birds, spectral sensitivities of UVS/VS photoreceptors peak at either 370nm (UVS) or 410nm (VS) [44]; this means that there are two types of colour vision in avian species: UVS or VS colour vision. We decided to objectively quantify spectral measurements obtained from both *D*. *hyparete* natural wings and the models’ artificial wings from an avian predator’s perspective using both types of avian vision. To do this, we analysed the wings’ reflectance spectra data using the R package Pavo [45], which uses avian visual sensitivities to estimate colour discriminability between models and real butterflies. Visual models as described by Vorobyev et al. [46] were used to determine colour distances with receptor noise based on relative photoreceptor densities of the default setting which is that of the blue tit *Cyanistes caeruleus* densities (1:2:2:4). We analysed reflectance spectra data through both UVS and VS visual systems [47] to obtain chromatic contrast values (Δ*S)*, which are given in units of “just noticeable difference” (jnd). By obtaining Δ*S values*, we are able to objectively describe the perceptual distance between two spectra from an avian vision perspective. There is controversy over the JND threshold with evidence suggesting that the threshold value is <1 [48]. However, past empirical studies have shown that, a JND value of less than three (< 3) indicates that birds are unable to differentiate between two spectra under normal viewing conditions [47, 49]. As previous work [50] suggested a threshold value of ≤ 3, we also adopted it as the criteria for our study.

### (d) Model preparation

**Fig 3:**
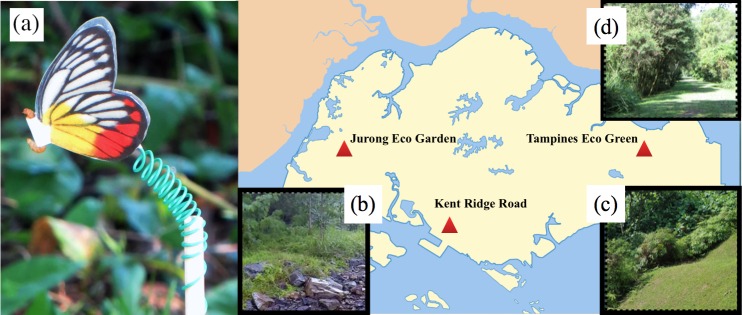
Photo of model in the field and localities of where field experiments were conducted. (a) Artificial butterfly model attached to live mealworm and a wooden rod via a coiled green metal wire. (b-d) The vegetation/general terrain found at each field site: (b) Jurong Eco Garden (Trial 3), (c) Kent Ridge Road (Trial 1), and (d) Tampines Eco-Green (Trial 2).

Each model was fitted with a live mealworm larva, *Tenebrio molitor*, using double coated tape (3MTM X-Series), and the model was attached to a wooden dowel rod through the use of a coiled green wire (Fig 3A). The mealworms were obtained from pet shops in Singapore. To prevent the mealworm from being attacked by ants or other crawling arthropods, we applied coats of insecticide (DIY Pest Control PC-CIDE) to the ends of the dowel rods as outlined by the Ho et al. study [43].

### (e) Field sites and experimental set-up

All predation experiments were carried out at three sites where *D*. *hyparete* can be naturally found, namely along Kent Ridge Road (01°17' N, 103°46' E), Tampines Eco Green (01°21' N, 103°56' E) and Jurong Eco Garden (01°21' N, 103°41' E) during the months of January and February of 2016 (Fig 3B–3D). We obtained permission from the National Biodiversity Centre, National Parks Board, Singapore, to perform these experiments at these locations. In these locations, the avian insectivores commonly observed were Javan mynas (*Acridotheres javanicus*), greater racket-tailed drongos (*Dicrurus paradiseus*), and zebra doves (*Geopelia striata*).

We placed all five types of models simultaneously at each of the three study sites to compare attack rates across all models under the same conditions. At each site we placed 100 models, 20 for each of the five patterns. A total of 300 models were used across the three sites. We used a smaller sample size in comparison to other predation studies on artificial butterfly models but a similar sample size (per model type) to that of Ho et al. [43] (Table 1). In addition, Ho et al. [43] had demonstrated that using live mealworm prey as a substitution for the butterfly’s body yielded fairly high attack rates in contrast to other substitutes (plasticine, clay) (Table 1). The models were placed two meters apart from each other, in clusters of five models, one from each pattern, with ten meters separating each cluster (S1 Fig). Models were left in the field for up to four days (96 hours) and checked daily for predation. A model was determined to have been predated/attacked when part of, or the entire mealworm had been eaten. If any of the treatment groups were observed to have half or more of its models predated (>10), we ended the experiment. This prevented over-estimating predation on the most aposematic models, which would be the majority of models remaining to be eaten, once most of the mealworm had been removed from the least aposematic models.

**Table 1:** Comparison of attack rates across different studies which used artificial butterflies’ paper models to study predator-prey interactions in field experiments.

Study	Type of model body used	Total no. of models used in study	Attack rate /%
Finkbeiner et al. [28]	Black Plasticine	1600	6.38
Ho et al. [43]	Live mealworm	720	54.4
Dell’aglio et al. [49]	Edible pastry body	608	19.2

### (f) Statistical analyses

To test for differences in predation rates, at each study site, between the control (Grey model) and each of the test models, or between two test models, we used Fisher’s exact probability test. The effect of wing colouration treatment across all three sites was analysed with a paired sample t-test, where number of predation events on the grey models and each of the test models were paired for each site. The same test was used to compare predation rates of two test models across the three sites. We also used a generalised linear mixed-effects model (GLMM) with a binomial distribution to test whether model colour affected probability of predation. Locality and number of days all models stayed in the field at each locality, were entered as random effects. Predation events were modelled as a binary response, with predated models assigned a value of 1 and non-predated models assigned a value of 0. Pair-wise comparisons to detect differences in predation probabilities were corrected using Tukey’s multiple comparisons method. All statistical analyses were performed using R Statistical Software.

### (g) Evolution of colour on the wings of *Delias* butterflies

To examine the evolution of wing colouration within the genus *Delias*, we scored photographs of both ventral and dorsal surfaces of 138 *Delias* taxa included on a species-level molecular phylogeny of *Delias* butterflies [30]. The phylogeny was based on three molecular markers: (i) cytochrome c oxidase subunit I *(COI)*, (ii) *wingless*, *and* (iii) elongation factor 1*α* (*EF-1 α*). The photos are available from an online museum database (Museum of Comparative Zoology, Harvard University) and the website http://www.delias-butterflies.com. Each butterfly surface was scored individually for the presence of yellow and/or red on the wings, regardless of the site and surface area of the colour. We then reconstructed the evolution of these colours on the phylogeny of *Delias* using parsimony and Mesquite software Version 3.04.

## Results

**Fig 4:**
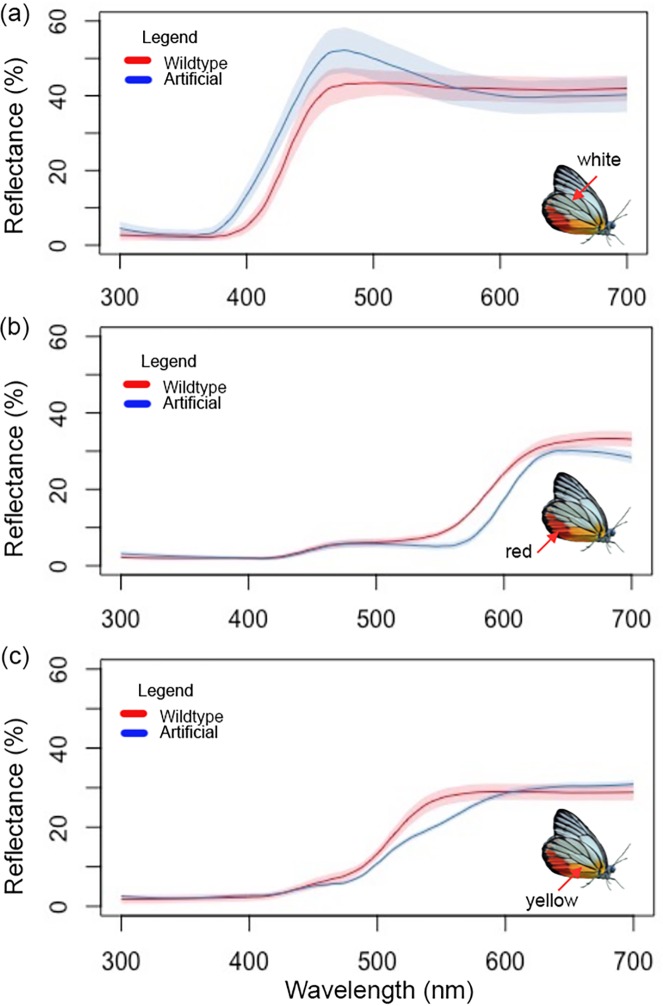
Plot of mean smoothed reflectance spectra of natural and artificial (paper) *Delias hyparete* hindwings. Shown are the mean values with shaded areas representing the standard deviation of the spectral data (n = 5 for each type) along with a ventral image of *D*. *hyparete* with an arrow indicating the colour that is being quantified through spectral measurements. Line colours for all three graphs indicate the specimen that is being measured, red: natural wings, blue: artificial wings. (a) White reflectance spectra, (b) Red reflectance spectra, and (c) Yellow reflectance spectra.

After optimising the colours of our models, the spectral analyses showed that the colours (white, red, yellow) on the paper models (Fig 4) are fairly similar to that of the natural butterfly wings from an avian perspective (Table 2). Achromatic contrasts were also calculated for the greyscale models (S1 Table). Multiple replicates of these models were thus used in our field experiments as proxies for the live butterflies.

**Table 2:** Chromatic contrasts values from colour discriminability calculations when spectral data was processed through avian visual systems (both UVS and VS).

	UVS			VS		
**JND comparisons**	*D*. *hyparete* white versus model white	*D*. *hyparete* red versus model red	*D*. *hyparete* yellow versus model yellow	*D*. *hyparete* white versus model white	*D*. *hyparete* red versus model red	*D*. *hyparete* yellow versus model yellow
Chromatic Contrast	2.1441	2.7602	2.0921	2.1441	2.2703	1.7566

Results are given in jnds, which describe the chromatic contrast between two spectra. A jnd value of < 1 suggests that models are indistinguishable by birds under normal viewing conditions, while values ≤3 indicates that the two colours under comparison are generally hard to distinguish from each other.

A total of 68 models, from the 300 placed in the field, showed signs of predation. Most models suffered attacks directed at the mealworm as well as adjacent areas of the paper wings (S2 Fig). Most predated models were observed to be still attached to the green wire with a few models found torn from the wire and left on the ground nearby. The green wires attached to most models were also stretched in either an upward or downward manner, which may be indicative of either an aerial or ground-based attack from the predators. From the attacked models, 29 were Grey models, 7 were Wt, 8 were Red, 13 were Yellow, and 9 were Blackless models. Even though only 23% of the models were attacked, rates of predation differed considerably between model types, with achromatic Grey models suffering 2 to 4 times higher predation than each of the other models.

### (a) Wild-type colouration of *D*. *hyparete* strongly deters predators

**Fig 5:**
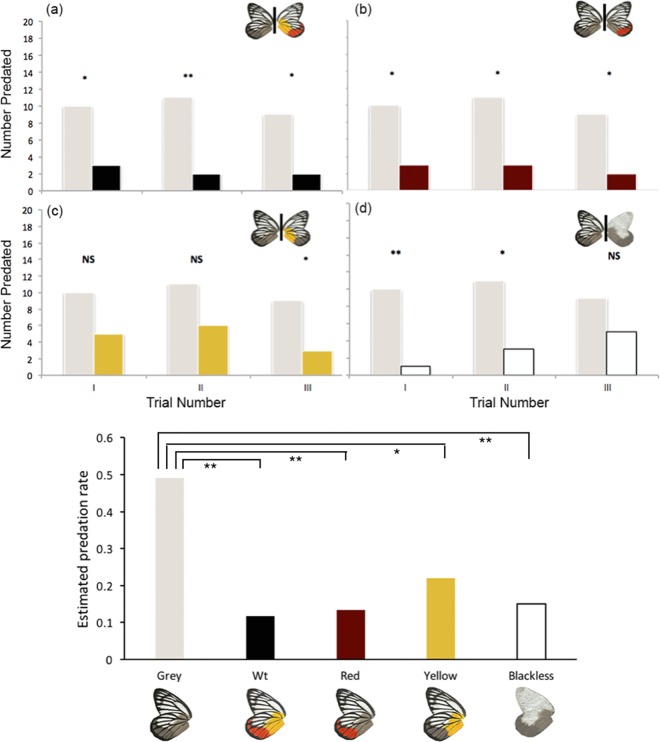
Number of predated test models relative to Grey models in each of the three trials and estimates of predation for each model type. (a-d) Asterisks represent the p-values from Fisher’s exact test (two-tailed) testing for differences in predation between the two model types. (e) Estimated predation rates for each model type across the three sites obtained from a generalised linear mixed-effects model (GLMM) analysis followed by post-hoc pair-wise comparisons (Tukey corrected). Only significant differences are indicated in the graphs. In both tests: *, p < 0.05, **, p < 0.01 and NS, not significant (p < 0.05).

Wt models were attacked significantly less relative to achromatic Grey models at each of the three sites (Table 3; Fig 5*A*). This difference remained significant when predation was assessed across the three sites (Table 4; Fig 5E).

**Table 3:** Number of days the models stayed in the field and Fisher's exact test (two-tailed) probability (*p*) for observed predation differences between control Grey model and each of the coloured models assuming no differences in signal effectiveness between the two models.

Coloured model	Trial	Number of days in field	*p*
**Wt**	1	3	0.0410*
	2	3	0.0057**
	3	4	0.0310*
**Red**	1	3	0.0410*
	2	3	0.0190*
	3	4	0.0310*
**Yellow**	1	3	0.1910
	2	3	0.1050
	3	4	0.0190*
**Blackless**	1	3	0.0033**
	2	3	0.0190*
	3	4	0.1050

*, *p* < 0.05

**, *p* < 0.01.

**Table 4:** Number of predation events for Grey and each of the coloured models across all three sites. t and *p* values (two-tailed) were calculated using paired sample t-tests. M and SD values denote mean predation and standard deviation across sites, respectively.

Coloured model	Trial	Number of grey models predated	Number of coloured models predated	t (df = 2)	*p*
**Wt**	1	9	3		
	2	11	2		
	3	9	2	8.3	0.0142*
**Mean**		9.67	2.33		
**Standard deviation**		1.15	0.58		
**Red**	1	9	3	12.1	0.0067**
	2	11	3		
	3	9	2		
**Mean**		9.67	2.67		
**Standard deviation**		1.15	0.58		
**Yellow**	1	9	5	8.0	0.0153*
	2	11	5		
	3	9	3		
**Mean**		9.67	4.33		
**Standard deviation**		1.15	1.15		
**Blackless**	1	9	1	5.0	0.0377*
	2	11	3		
	3	9	5		
**Mean**		9.67	3		
**Standard deviation**		1.15	2		

### (b) Red is an effective warning signal

Similarly, Red models were significantly less predated at each of the sites relative to Grey models (Table 3; Fig 5*B*). The difference remained significant when assessed across the three sites (Table 4; Fig 5E). The number of attacks on these models was similar to those towards Wt models (paired T-test: t = 1.0, p = 0.423, Table 5).

**Table 5:** Test statistics for post-hoc pairwise comparisons of predation probability across model types (using Tukey’s correction) after running a generalised linear mixed effects model (GLMM) analysis with binomial distribution, logit link function, where model type was used as a fixed variable and locality and duration of each experiment were used as random variables.

**Model Comparisons**	**Estimate (Means)**	**Standard Error**	**Z value**	**P value (>|z|) (Tukey corrected)**
**Blackless-Grey**	-1.70	0.45	-3.82	0.001*
**Red-Grey**	-1.84	0.46	-3.99	<0.001**
**Wt-Grey**	-1.99	0.48	-4.16	<0.001**
**Yellow-Grey**	-1.23	0.41	-3.01	0.021*
**Red-Blackless**	-0.14	0.52	-0.26	0.999
**Wt-Blackless**	-0.29	0.54	-0.54	0.983
**Yellow-Blackless**	0.47	0.48	0.98	0.861
**Wt-Red**	-0.15	0.55	-0.28	0.999
**Yellow-Red**	0.61	0.49	1.23	0.729
**Yellow-Wt**	0.76	0.51	1.49	0.565

### (c) Yellow also serves as an effective warning signal

Yellow models suffered lower predation relative to Grey models at each of the three sites, but the difference in predation events was significant only at the third site (Table 3; Fig 5*C*). Data pooled across the three sites, however, showed Yellow models suffering significantly less predation than Grey models (Table 4, Fig 5E), and did not differentiate predation rates on these models relative to Wt models (paired T-test: t = 2.65, p = 0.12, Table 5).

### (d) Absence of black venation patterns leads to decreased predation

Blackless models suffered fewer attacks relative to Grey models containing black veins in all three sites, and two of three trials showed the differences to be significant (Table 3; Fig 5*D*). The difference remained significant when results from all three trials were pooled together (Table 4, Table 5, Fig 5E).

### (e) Colours that function as warning signals are predominantly found on the ventral surface of *Delias* wings

The experiments above indicate that red and yellow colours both serve as aposematic signals in *Delias hyparete* with equal efficacy, and black does not serve an aposematic function. As the colour patterns of *Delias* species are mainly composed of black, yellow, and red colour patches on a white background, we explored the evolution of the two aposematic colours, red and yellow, on a phylogeny of *Delias* butterflies, scoring exclusively male wing colour patterns.

**Fig 6:**
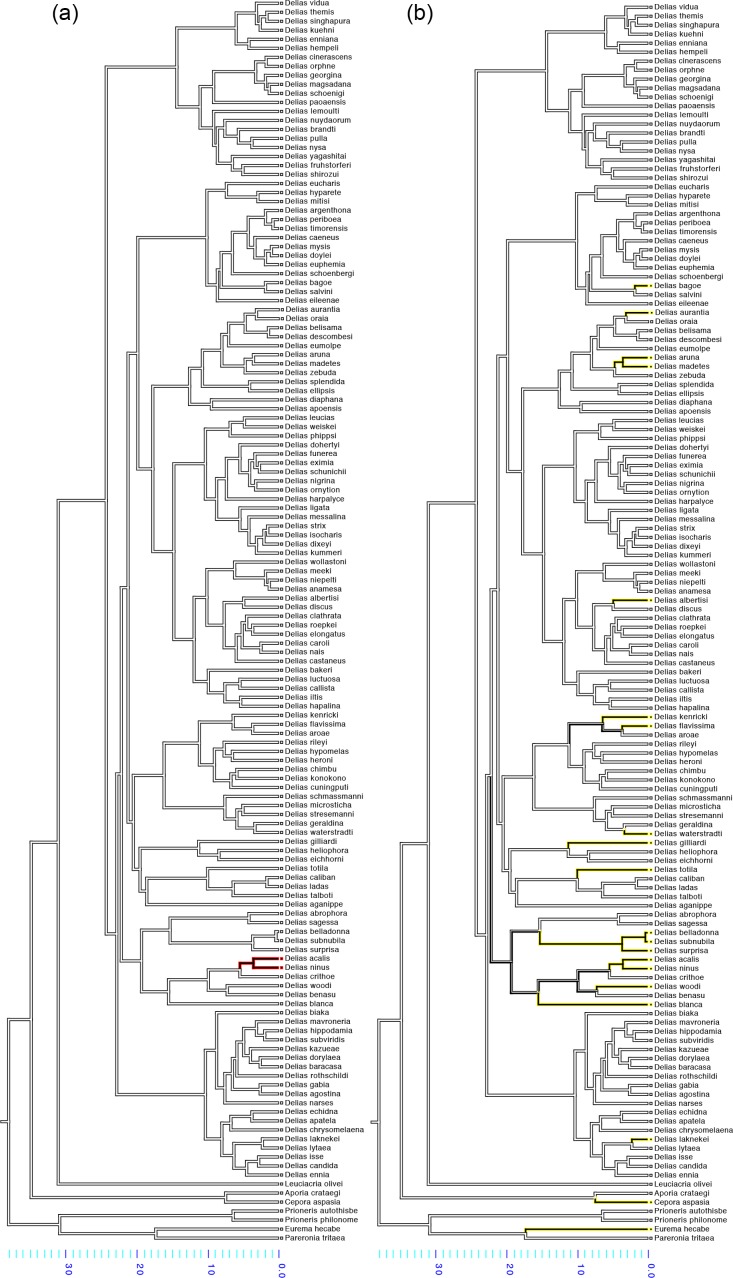
Parsimony reconstruction of the evolution of dorsal colour amongst 138 species of *Delias* using the molecular phylogeny of Muller et al. (2013). (a) Red colour evolution. (b) Yellow colour evolution. Branches are coloured as follows: yellow–yellow colour is present; red–red colour is present; white–white with black patterns are present. Only male specimens are considered in this study.

Male dorsal wings are mostly white and black (Fig 6). Out of the 138 taxa scored, 122 do not have any red or yellow markings on the dorsal surface. Yellow colouration has evolved independently on the dorsal wings at least 15 different times, with a single instance of both red and yellow colours evolving concurrently in the last common ancestor of *Delias acalis and Delias ninus* (Figs 6 and S3).

**Fig 7:**
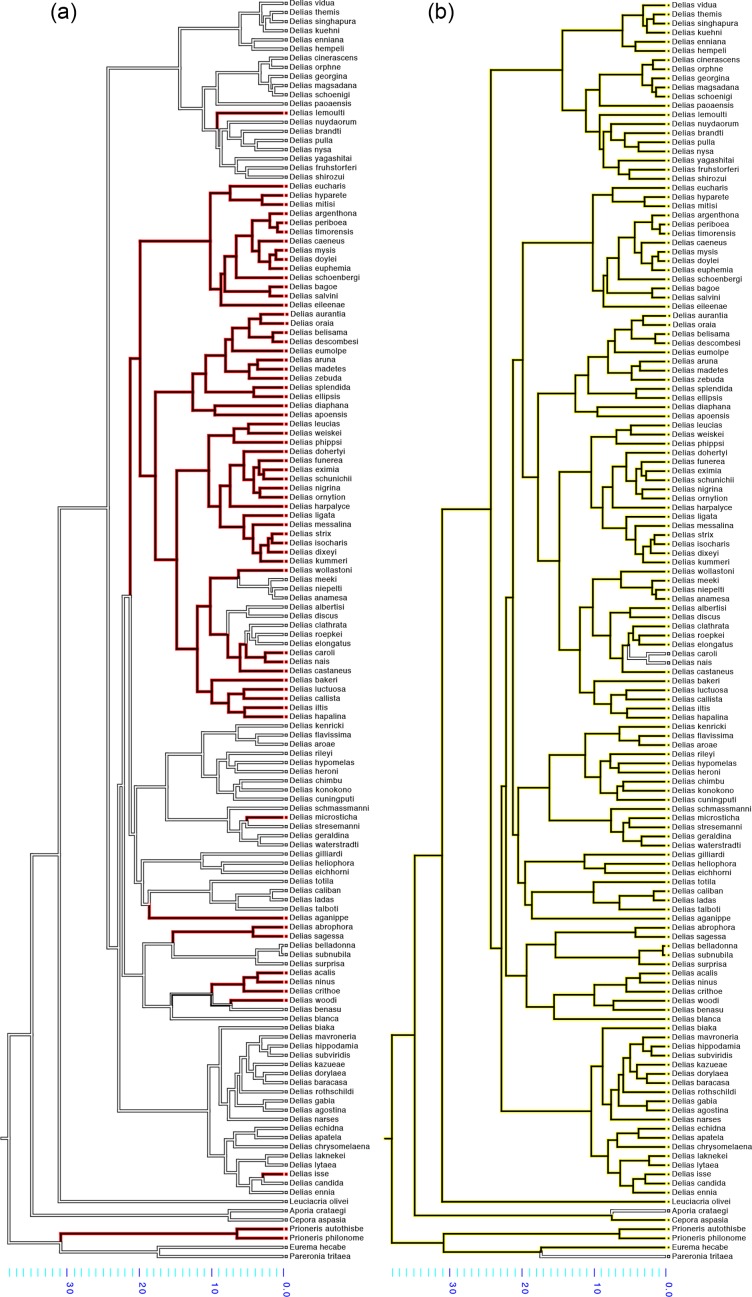
Parsimony reconstruction of the evolution of ventral colouration amongst 138 species of *Delias* using the molecular phylogeny of Muller et al. (2013). (a) Red colour evolution. (b) Yellow colour evolution. Branches are coloured as follows: yellow–yellow colour is present; red–red colour is present; white–white with black patterns are present. Only male specimens are considered in this study.

In contrast, with the exception of 2 outgroup species (*Aporia crataegi* and *Pareronia tritaea)*, all *Delias* species included in the phylogeny had either red, or yellow, or both colours present on their ventral wings (Fig 7). Yellow is reconstructed as an ancestral colour for the genus while red is reconstructed as a derived colour, only appearing in a sub-set of the clades. In these derived clades there are three cases of reversions involving loss of red pigmentation. These involve an ancestor species with red and yellow colouration yielding daughter species with just yellow colours on their ventral wings (Figs 7 and S4). In general, however, ventral colouration evolved from a pattern containing only yellow patches to patterns containing additional red patches, with few to no colour reversals.

## Discussion

We explored the hypothesized aposematic function of *D*. *hyparete* colouration by testing the efficiency of its ventral colour patches in deterring predator attacks. Our results clearly show that the wild-type colouration of the Painted Jezebel deters predation. The behaviour of predators towards the models may be due to innate avoidance towards prey with these colour patterns, or due to a negative prior experience with a real prey. As the field experiments were conducted in localities where *D*. *hyparete* is naturally found, it is likely that the predator community had prior experience of the wild-type butterfly, however, innate effects could also be playing a role. Regardless of mechanism, our results suggest that the colouration of the Painted Jezebel functions as an aposematic signal.

By comparing the predation of models faithfully resembling the Wt colour pattern with that of models with parts of the colour pattern replaced by a grey shade, our study tried to address which of the colours of the Painted Jezebel was producing the aposematic response. We found that not all colours were equally efficient in deterring predation. At least for the predator community tested in this study, red and yellow seem to represent a more effective warning signal than black.

Higher signal stability, as well as contrast, of both the Red and Yellow models might explain why they performed better in field predation tests as compared to Grey models in our study. Higher signal stability may be due to the fact that both red and yellow are colours with longer wavelengths and thus, are less susceptible to scattering by atmospheric particles. As such, they are perceived as more uniform and stable colours across the time of day [51]. Atmospheric particles and cloud cover tend to scatter shorter wavelengths through the process of Rayleigh scattering, thus negatively affecting the stability of a signal [51]. In addition, signal contrast against the natural habitat of the prey, which tends to be green vegetation, is theorized to contribute to aposematic signal effectiveness [52]. Both red and yellow are perceived as having higher contrast against green backgrounds as compared to other colours. For instance, when ladybird colouration was modelled to an avian visual system and the contrast of the ladybird’s colouration was measured against an average green background, red colours remained extremely stable throughout the day, unaffected by the varying illuminant spectra caused by differences in time of day and atmospheric conditions. Although yellow colours were found to not be as salient as red over varying light conditions, contrast values of yellow stimuli studied in the aforementioned study still revealed yellow to be a contrasting colour against natural backgrounds [51].

Physiological mechanisms that explain why certain longwave colours such as red are more highly contrasting against green have to do with how colours are perceived in the retina and interpreted by the brain. In humans, colour vision is reliant on the selective activation of three different types of cone receptors located in the retina, and also on the presence of three opponent mechanisms which are receptor complexes responsible for sensing antagonistic pairs of colours such as blue–yellow, red–green, and black–white [53]. In other words, when the eye is detecting such colour pairs, the neural pathways that are associated with each colour within a pair will be processed in an antagonistic fashion. That is, if a red stimulus is received by photoreceptors in the predators’ eyes, it will be perceived as an especially contrasting colour against green after getting processed through the red–green opponent system. Likewise, a study of domestic chicks (*Gallus gallus*) discovered the existence of at least three colour opponent channels that function in a similar fashion to those of humans [46]. If birds are the primary predators of *Delias* butterflies in the wild, this conserved physiological system of colour perception should have been effectively exploited by *Delias* butterflies, which should have gradually evolved more effective, stable, and contrasting aposematic signals.

Consequently, if both red and yellow are effective as warning signals, and if these colours are functioning exclusively as aposematic signals, we expected that both colours, once evolved, should show few evolutionary reversals. This was supported by our reconstruction of ventral colour evolution on the *Delias* phylogeny (Fig 7A), which only showed three instances of red colour reversions and two instances of yellow colour reversions (S4 Fig). Yellow was found to be an ancestral colour, with red being a more derived colour in later lineages. Of the lineages that lost yellow, one was an outgroup species to the *Delias* genus while the other two species replaced yellow for red colouration. Although our study has shown no significant difference in predation between Red and Yellow model types of *D*. *hyparete*, we propose that warning signal efficacy may have evolved gradually within the genus *Delias*, with red being a derived and slightly more effective aposematic color. However, it should be noted that our phylogenetic analysis of the *Delias* butterflies is not exhaustive. If possible, more molecular data should be retrieved from additional *Delias* species for a more accurate sampling of the evolution of the ventral and dorsal colouration. Likewise, larger studies with the same or other members of the genus should be carried out to test whether red signals confer more protection upon *Delias* adults as compared to yellow signals, over a range of lighting environments, as they are predicted to do.

Unlike ventral colouration, the general lack of either red or yellow colouration on the dorsal wings of *Delias* species suggests that these wing surfaces might not play a role in aposematic signalling, and in signalling to predators in general. Studies of other butterfly genera, such as those of the family Nymphalidae, have shown that dorsal wing patterns function primarily as sexual signals [54–56] whereas ventral patterns mostly aid in deterring predation [43, 57, 58]. The most likely explanation for the signal partition between dorsal and ventral surfaces is that, barring those species that upon sensing danger have a wing flashing display [59], most butterflies fold and hold their wings over their body, and primarily expose their ventral wing surfaces even during an attack. Thus, by evolving conspicuous colours on the ventral surfaces, aposematic butterflies will gain a considerable selective advantage, as it is more likely that predators will be able to spot the distasteful butterfly from a distance, associate the colours with unprofitability, and be deterred from launching an attack.

Dorsal surfaces of *Delias* butterflies are mostly white and black. A previous study showed that females of another pierid butterfly, *Pieris rapae*, with similar dorsal colouration to *D*. *hyparete*, prefer “chromatic” males with bright white colouration in the long wavelengths which are dark in the UV wavelengths [60]. Thus, it is possible that in *Delias* adults, the bright white dorsal colouration, containing little UV signal, might be an equally important signal in mate choice.

Whether colours such as red or yellow present on the ventral surface also function in sexual signalling warrants additional work, as studies have demonstrated that two other pierid butterflies might have the potential to visually discriminate the colour red. *Colias erate*, the Eastern Pale Clouded Yellow, was discovered to be sensitive to red colour by creating red channels through the novel process of using red screening pigments to act as selective filters on existing photoreceptors. These visual pigments are sexually dimorphic with females having three more channels with red wavelength sensitivity as compared to males [61]. Moreover, the Small Cabbage White, *Pieris rapae*, also has varying shades of red clustered pigments present in the ommatidia, which serve as filters to produce photoreceptors with peak sensitivities at either 620 or 640nm [62]. Although it is theoretically possible that these butterflies are able to discriminate colours within the red range of wavelengths, confirmatory behavioral experiments are lacking.

Lastly, our study indicated that models without the black veins and overall grey colouration were significantly less predated than models with the black veins (Grey models). This was unexpected as black is generally described as an aposematic colour in the literature [10]. One possible reason for this observation is that different components of a colour pattern serve varying roles. While red and yellow may signal unprofitability to a potential predator, black veins may serve to increase the salience of the white, red, and yellow colours [12]. Black outlined colours would help to attract the attention of a predator, improve signal recognition, and also accelerate avoidance learning. It might be plausible that the white colour of the models might also serve as an aposematic signal, explaining the decreased rate of predation of Blackless models, which increased in their white coloured area. However, a previous study conducted by Lyytinen et al. [63] suggested that the white colouration of pierid butterflies are unlikely aposematic signals because predators in their study attacked both white and non-white butterflies at similar rates. Thus, the reason why Blackless models experienced significantly less predation relative to Grey models could simply be due to the predators being unable to detect them in the first place.

## Conclusion

We have shown that red and yellow colours serve as warning signals for the Painted Jezebel butterfly, *D*. *hyparete*, and protect this butterfly from predation. Our phylogenetic analyses also showed that red is a novel colour that originated within the genus and that both red and yellow are more widespread on ventral surfaces, which suggests that ventral characters are responsible for advertising warning signals as compared to dorsal colouration in *Delias*.

As the world’s largest butterfly genus, the colouration of *Delias* is extremely variable and yet restricted to just permutations of red, yellow, white and black colours. Having demonstrated the aposematic function of warning colouration in *Delias*, future research could focus on selective pressures other than predation that might also be responsible in driving the evolution of bright signals in *Delias*. In addition, future work could concentrate on resolving the proximate mechanisms underlying the evolution of colour and patterns across *Delias* species. In addition, biochemical characterization of the compounds found in the bodies of these butterflies, together with toxicity bioassays, should be carried out to test whether *Delias* species are truly aposematic, or whether certain clades are Batesian mimics of closely related toxic species.

## Supporting Information

S1 Fig Visual representation of how models were placed in the field at each site. Shown here are two sets of five models placed in the field. The models are randomly placed within each set with a distance of two meters separating each model. Each set is spaced 10 meters away from each other.
(TIF)

S2 Fig Some examples of the artificial paper models that had suspected bite marks from predators. These are examples of models found tore from the wooden rods and dropped near the vicinity of the rods.
(TIF)

S3 Fig **Dorsal view of (a) *Delias acalis*, and (b) *Delias ninus*** the only two species in our phylogenetic treatment that display red pattern on the dorsal surface. (Source: Museum of Comparative Zoology, Harvard University and the Smithsonian Tropical Research Institute)
(TIF)

S4 Fig Close-up of the single *Delias* clade where red and yellow colours were lost. The ancestral colour reconstructions indicate three single losses of red colours (denoted by red star markers) from an ancestral species that had both red and yellow colours on its ventral wing.
(TIF)

S1 Table Achromatic contrast values of greyscale models as modelled through the vision of the blue tit *Cyanistes caeruleus* Results are given in jnds, which describe the chromatic contrast between two spectra. A jnd value of < 1 suggests that models are indistinguishable by birds under normal viewing conditions, while values ≤3 indicates that the two colours under comparison are generally hard to distinguish from each other.
(TIF)
