# Supplementary figures and images for: Yellow and the Novel Aposematic Signal, Red, Protect Delias Butterflies from Predators
Source: PLoS One. 2017 Jan 6;12(1):e0168243. doi: 10.1371/journal.pone.0168243 (PMC5218396; doi:10.1371/journal.pone.0168243)

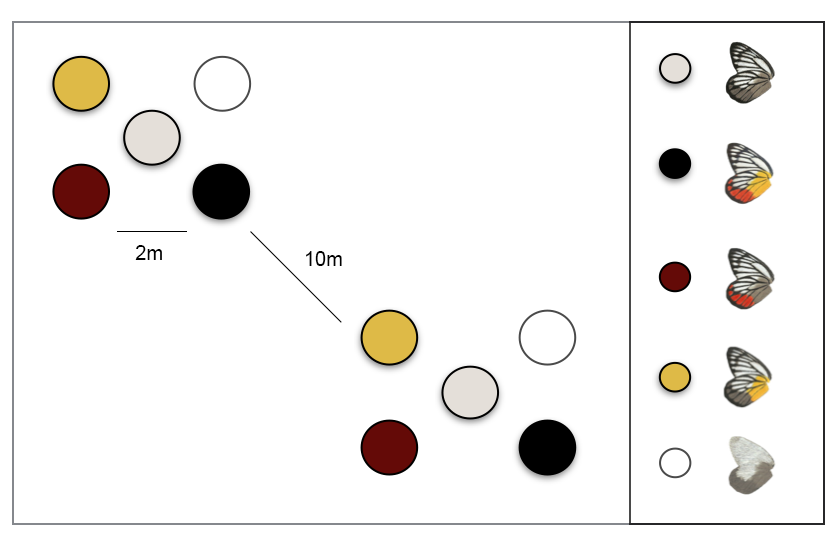

Supplement: S1 Fig — Shown here are two sets of five models placed in the field. The models are randomly placed within each set with a distance of two meters separating each model. Each set is spaced 10 meters away from each other. (TIF) [file pone.0168243.s001.tif]

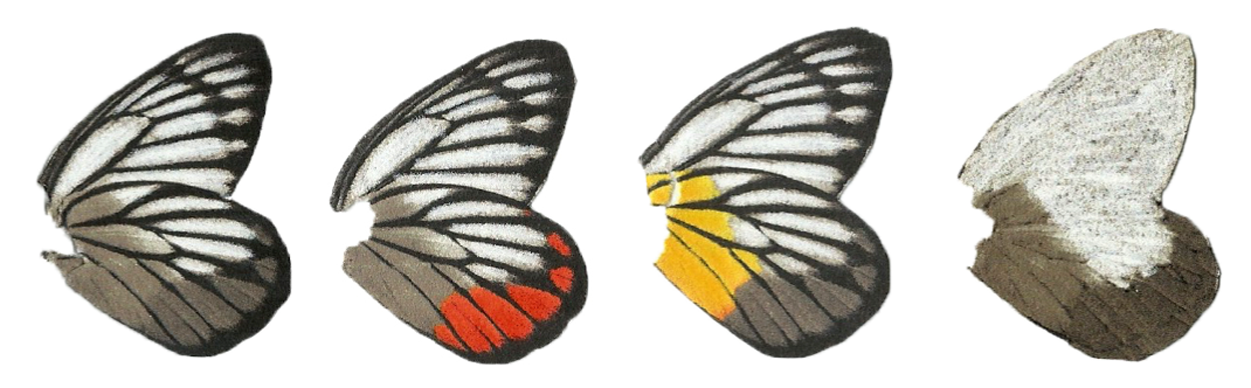

Supplement: S2 Fig — These are examples of models found tore from the wooden rods and dropped near the vicinity of the rods. (TIF) [file pone.0168243.s002.tif]

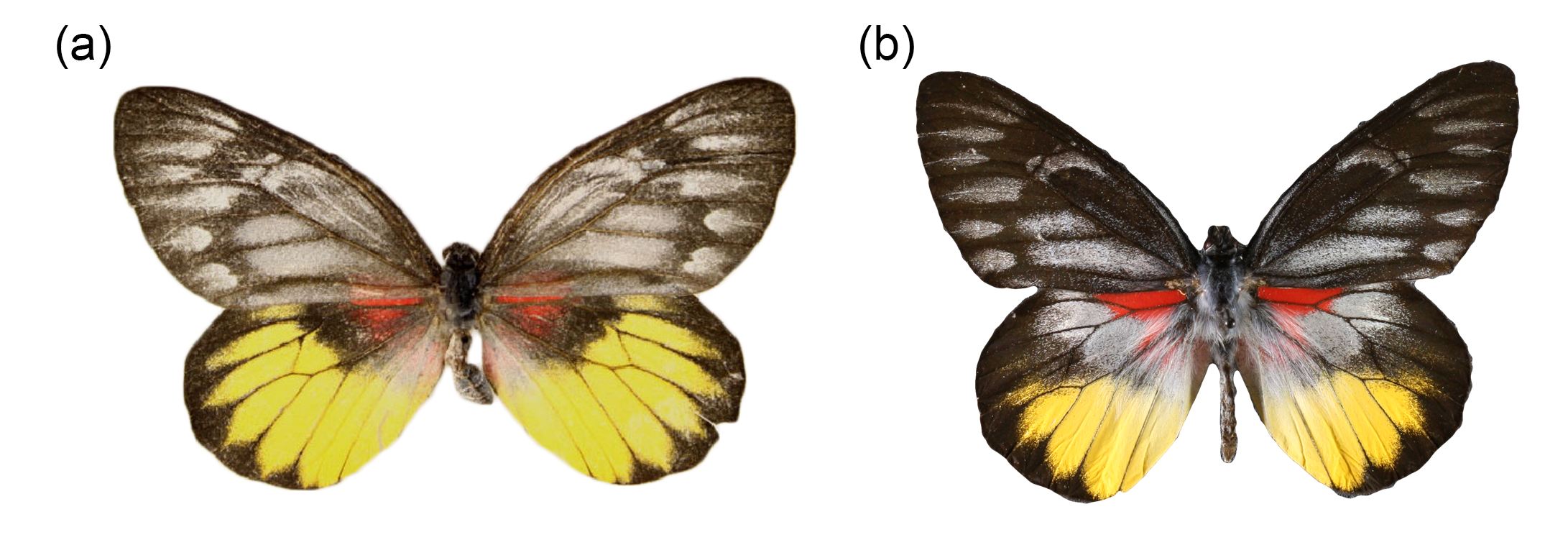

Supplement: S3 Fig — Dorsal view of (a) Delias acalis, and (b) Delias ninus the only two species in our phylogenetic treatment that display red pattern on the dorsal surface. (Source: Museum of Comparative Zoology, Harvard University and the Smithsonian Tropical Research Institute) (TIF) [file pone.0168243.s003.tif]

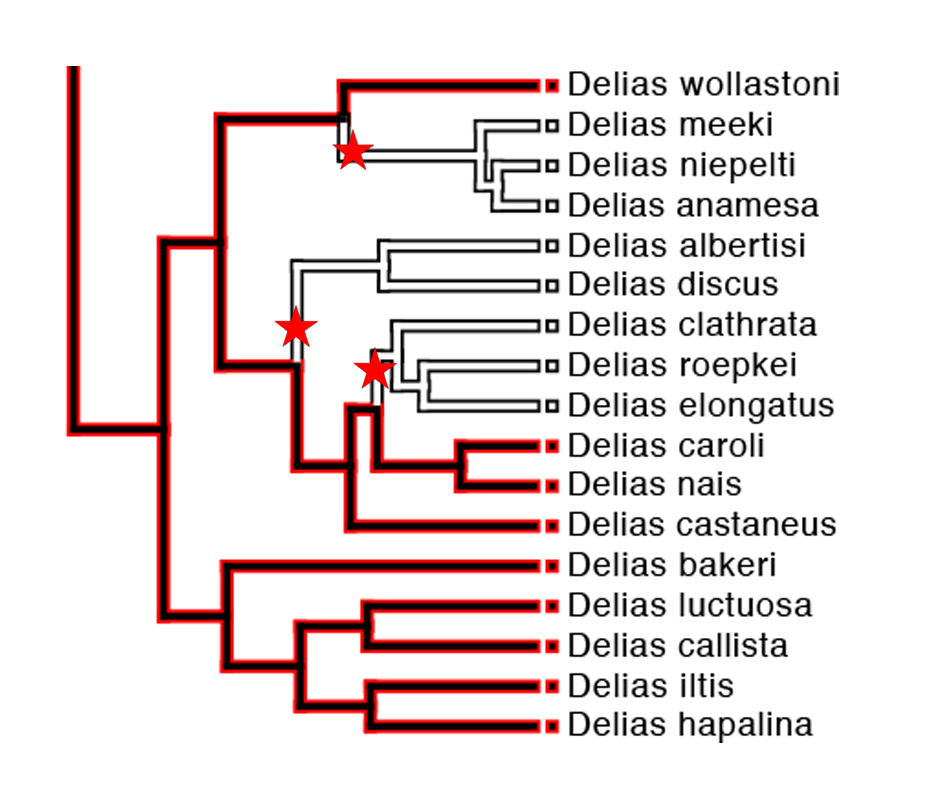

Supplement: S4 Fig — The ancestral colour reconstructions indicate three single losses of red colours (denoted by red star markers) from an ancestral species that had both red and yellow colours on its ventral wing. (TIF) [file pone.0168243.s004.tif]

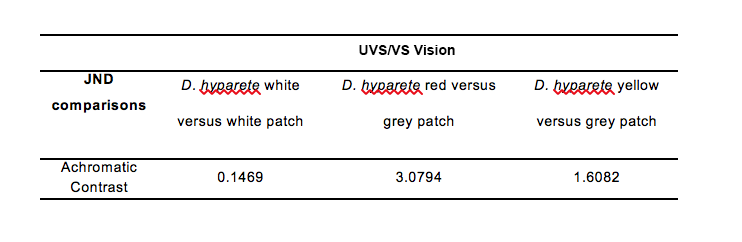

Supplement: S1 Table — A jnd value of < 1 suggests that models are indistinguishable by birds under normal viewing conditions, while values ≤3 indicates that the two colours under comparison are generally hard to distinguish from each other. (TIF) [file pone.0168243.s005.tif]
